# Molecular docking analysis of Enterotoxin I from Staphylococcus aureus with Nafcillin analogues

**DOI:** 10.6026/97320630016731

**Published:** 2020-10-31

**Authors:** Jayaraman Selvaraj, Rajagopal Ponnulakshmi, Veeraraghavan Vishnupriya, Chandrasekar Kirubhanand

**Affiliations:** 1 Department of Biochemistry, Saveetha Dental College and Hospitals, Saveetha Institute of Medical and Technical Sciences, Saveetha University, Chennai - 600 077, India; 2 Central Research Laboratory, Meenakshi Academy of Higher Education and Research (Deemed to be University), West K. K. Nagar, Chennai-600 078, India; 3 Department of Anatomy, All India Institute of Medical Sciences (AIIMS), Nagpur, India

**Keywords:** S *aureus*, Nafcillin, enterotoxins I, molecular docking

## Abstract

Staphylococcal enterotoxins (SEs) are liked with food poisoning and other related infections. Nafcillin is an antibiotic used to treat S. aureus. Therefore, it is of interest to study the molecular interactions of 25 nafcillin analogues with enterotoxin I using molecular docking analysis. The analysis shows optimal interaction features of Nafcillin analogues with Enterotoxin I from Staphylococcus aureus for further consideration.

## Background:

Staphylococcus aureus (S. aureus) is a gram-positive bacterium that causes a broad range of diseases from mild skin infections to life- threatening diseases such as necrotizing pneumonia and bacteremia. S. aureus infection is one of the rising alarms for family physicians, because of its high occurrence of morbidity and antimicrobial resistance. S. aureus virulence capacity mainly relies on the production of a notable list of protein toxins. These are able to work alone or in concert to cause a multitude of human being diseases [1]. Enterotoxins are short secreted proteins, soluble in water and saline solutions. These Enterotoxins have common structural and biochemical features and it's extremely resistant to heat. Prolonged boiling or autoclaving can reduce the effectiveness of these types of proteins. They are extremely resistant to the majority proteolytic enzymes, and thus retain their action in the digestive tract after ingestion [2]. Pneumonia, the sepsis-related infections, toxic shock syndrome, and food poisoning are some of main diseases associated with enterotoxins [3]. There are so many recent reports proposed that the staphylococcal enterotoxins (SEs) play a wider part in the manifestation of other human diseases like the diseases connected to the respiratory tract [4,5] and autoimmune disorders [6,7]. The S. aureus enterotoxins are potent non-specific T-cell stimulators (super antigens) involving the unregulated activation of the immune response [8]. A huge cytokine load is formed creating the clinical features of toxic shock syndrome, which is linked to fever, organ malfunction leading to significant mortality [9].

Compared to other toxins produced by S. aureus, enterotoxins needs only tiny amount to be toxic in human. So this enterotoxin can act as potential target for the identification of new drug candidate against S. aureus infections. The treatment of bacterial infections has become gradually more difficult because of the emergence of antibiotic resistance. Symbolic of these issues are strains of methicillin-resistant Staphylococcus aureus (MRSA) that have attained epidemic in mnay countries [10 11]. In the United States, S. aureus is the frequent source of hospital and community-associated bacterial illness of the bloodstream, skin and soft tissue and other sites, with MRSA strains containing a huge majority in lots of locales [12,13]. The occurrence of S. aureus infections is increasing. Antimicrobial agents such as daptomycin, linezolid and nafcillin or oxacillin are used for treatment. Therefore, it is of interest to study the molecular interactions of 25 nafcillin analogues with enterotoxin I using molecular docking analysis.

## Materials and Methods:

### Structure of Enterotoxin I protein:

The structure of Enterotoxin I protein (PDB ID: 2G9H) [14] was downloaded from the Protein Data Bank (PDB) and processed using the CHARMM force field using standard procedures.

### Ligand Preparation:

The 2-D structure of Nafcillin and its analogue structures were downloaded from pubchem database [15]. These 2-D structures were converted as 3-D structures using the Online Smiles Translator. The compounds were processed using the CHARMM force field following standard procedures. Lipinski's properties like molecular weight, log P and number of Hydrogen-bond donors and acceptors for the active compounds were calculated and validated.

### Active site prediction:

Active site residues in the enterotoxins were predicted using discovery studio 2.1.

### Molecular docking:

Molecular docking analysis of enterotoxins I and the Nafcillin analogues were completed using the Lib dock module in Discovery studio (Version 2.1, Accelry's Software Inc.) [16].

## Results and Discussion:

Molecular Docking:

Interaction between protein and ligand plays considerable role in structure based drug-designing approach. In the present study discovery studio 2.1 was used to perform the docking calculation. Based on the scoring functions discovery studio evaluate the binding affinity and their stability of the docked complexes. Before performing docking studies the binding site of enterotoxin I protein was predicted through Discovery studio 2.1. The following residues THR 80, THR 74, ASN 15 were present in the predicted as active site pocket. The purpose of this study was to use LibDock program to estimate the binding capacity between the protein enterotoxin I and the compounds nafcillin and its analogues. A total of 25 analogues were selected for the study. The structure of enterotoxin I was used for the docking study. Many conformations were produced using docking. Based on the scoring parameters only top ranked complexes were selected for further binding affinity analysis.

LibDock score was applied which is PLP like score (Steric and H-bonding intermolecular functions) to link the biological activity of the protein and ligands,. Higher PLP scores assign stronger protrein ligand binding [17, 18]. These scoring functions have helped to identify the active and inactive compounds. To make sure that the ligand orientations attained from the docking studies were probable to signify suitable and sensible binding modes, the LibDock program-docking parameters had to be verified in the crystal structure’s active site. Utilities in Discovery's studio help to ascertain the binding site of protein structure. Results of docking confirmed that LibDock find the most favorable orientation of the docked compound accurately to the active sites. Both nafcillin and its analogues bind to the same amino acids residues in the active site region.

**Figure 1:**
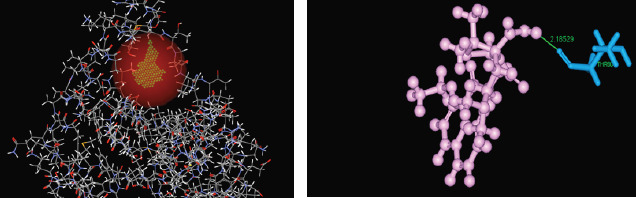
Molecular interaction of nafcillin with enterotoxin I

**Figure 2:**
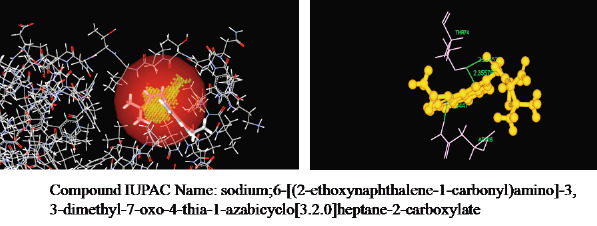
Molecular interaction of the best analogues 1 with enterotoxin I

**Figure 3:**
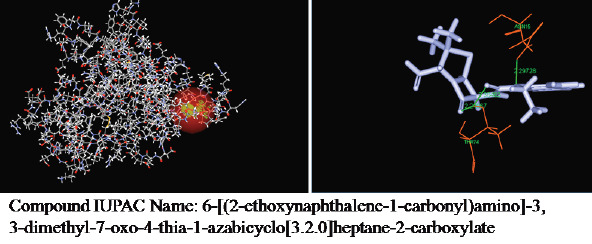
Molecular interaction of the best analogues 2 with enterotoxin I

LibDock score of nafcillin is 99.525, the hydrogen bond length is 2.1Å and energy values 96.128. Amino acid involved in the hydrogen bond formation [THR-80] is noted. Out of 25 analogues two analogues were selected based on the scoring parameters. A LipDock score of two analogues are 101.494 and 100.608 respectively. The energy values also occur close the original compounds nafcillin. The energy vales of two best analogues are 97.664 and 77.56 (Table 1 - see PDF). Analysis of docking results showed that the hydrogen bonding interactions between THR74 and ASN 15 mainly contributed the compounds and target protein. These two residues alternatively form the H-bond with most of the compounds. So it plays the functional role of the protein. The analysis of the best docked ligands allowed the binding mode of compounds involved in this study and confirmed the role as anti-bacterial agent. The overall structure obtained by docking of Nafcillin to enterotoxin I was given in Figure 1. Interaction of other analogues with enterotoxin was also shown in Figure 2-Figure 3.

## Conclusion

We document the optimal interaction features of 25 Nafcillin analogues with Enterotoxin I from Staphylococcus aureus for further consideration.
